# Hereditary colorectal cancer and Lynch syndrome

**DOI:** 10.1186/1753-6561-7-S2-K2

**Published:** 2013-04-04

**Authors:** Benedito M Rossi

**Affiliations:** 1 Hospital de Cancer de Barretos, Barretos, São Paulo – Brazil

## 

The term Hereditary Nonpolyposis Colorectal Cancer, or HNPCC, has been less used for naming the classical autosomal dominantly inherited susceptibility to cancer [1]. As this susceptibility applies to tumors from different primary sites other than but including colorectal cancer (CRC), the term Lynch Syndrome (LS) is a less restrictive name. Lynch Syndrome is characterized by an autosomal dominantly inherited susceptibility to cancer, caused by inherited germline mutations in the mismatch repair (MMR) genes. It is characterized by early age of onset, predilection to the proximal colon, multiple primary CRCs, and extracolonic tumors, particularly endometrium carcinoma (EC) [2,3].

To establish a profile of the disease, a better definition of the spectrum of related tumors has been a constant concern [4,5]. It is therefore expected to find heterogeneity among the families regarding the susceptibility to develop tumors in different specific sites. The risk of cancer in fact varies among families with LS, although the variation does not necessarily result from genetic heterogeneity. The standards of environmental exposure must contribute to the differential gene expression, justifying at least in part this heterogeneity [6].

LS accounts for 2%–5% of all CRC cases [7]. In fact, it is believed that 20% to 30% of patients with CRC present some type of genetic susceptibility, but without meeting criteria for known typical syndromes. However, new cancer cases in the patient’s family or supplemental information on previously unknown cases can lead to a reclassification that may characterize a typical syndrome. In other situations, despite the lack of clinical criteria for determining an inherited character, molecular inquiry can define the diagnosis of inherited syndrome. For these reasons, even in the absence of typical clinical characterization, criteria must be used to direct the inquiry of an inherited condition.

The spectrum of extracolonic tumors in LS began to be the subject of several publications in which the most common cancers found were those affecting the endometrium, the stomach and the urinary tract [8-14]. Watson and Lynch [6] calculated the frequency of cancer in other specific sites in 1,300 high-risk individuals from 23 families having LS and demonstrated a significant increase of the risk of developing cancer in the stomach (RR:4.1), small bowel (RR:25), kidneys (RR:3.2), ureter (RR:22), and ovary (RR:3.5). The proposed extracolonic cancers associated with LS are endometrium, stomach, ovary, small bowel, ureter, renal pelvis, brain, and hepatobiliary tract. Among these tumors, endometrium, ureter, renal pelvis, and small bowel cancers present the highest relative risk, and are therefore the most specific for LS.

The Amsterdam criteria for clinical diagnosis of LS are: (1) at least three relatives must have histologically verified CRC, endometrium, ureter, renal pelvis, or small bowel cancer; (2) one must be a first-degree relative of the other two; (3) at least two successive generations must be affected; (4) at least one of the relatives with cancer must have received the diagnosis before age 50; and (5) familial adenomatous polyposis must have been excluded. Because there are families with an MMR mutation present exclusively in patients with endometrial cancer without CRC, the requirement of at least one case of CRC was suppressed [15,16].

Patients with LS may also have sebaceous adenomas, sebaceous carcinomas, and multiple keratoacanthomas, findings consonant with Torre’s syndrome variant [17,18]. The definition of LS includes a familial clustering of colorectal and/or endometrial cancer and as associated cancers stomach, ovary, ureter/renal pelvis, brain, small bowel, hepatobiliary tract, and skin (sebaceous tumors) tumors [15,19].

As discussed by Vasen [20], since it is known that LS is caused by an mismatch repair gene defect and that the hallmark of the syndrome is microsatellite instability (MSI), more attention should be given to the so-called Bethesda guidelines, which describe almost all clinical conditions in which there is suspicion of LS and in which a search for MSI is indicated, mainly early onset of colorectal adenomas and cancer.

## Competing interests

There are no competing interests in this presentation.
